# Influence of psychopathology and metabolic parameters on quality of life in patients with first-episode psychosis before and after initial antipsychotic treatment

**DOI:** 10.1038/s41537-023-00402-8

**Published:** 2023-11-07

**Authors:** Anne Sofie A. Dahl, Victor Sørensen, Karen S. Ambrosen, Mikkel E. Sørensen, Grímur H. Mohr, Mette Ø. Nielsen, Kirsten B. Bojesen, Birte Y. Glenthøj, Margaret Hahn, Julie Midtgaard, Bjørn H. Ebdrup

**Affiliations:** 1 Center for Clinical Intervention and Neuropsychiatric Schizophrenia Research (CINS) & Center for Neuropsychiatric Schizophrenia Research (CNSR), Copenhagen University Hospital—Mental Health Services Copenhagen, Copenhagen, Denmark; 2 Centre for Applied Research in Mental Health Care (CARMEN), Copenhagen University Hospital—Mental Health Services Copenhagen, Copenhagen, Denmark; 3 Department of Clinical Medicine, Faculty of Health and Medical Science, University of Copenhagen, Copenhagen, Denmark; 4 Schizophrenia Division, Centre for Addiction and Mental Health (CAMH), Toronto, ON Canada; 5 Institute of Medical Science, Temerty Faculty of Medicine, University of Toronto, Toronto, ON Canada; 6 Department of Psychiatry, University of Toronto, Toronto, ON Canada; 7 Banting and Best Diabetes Centre, University of Toronto, Toronto, ON Canada

**Keywords:** Human behaviour, Psychosis, Schizophrenia

## Abstract

The impact of psychological and physical health on quality of life (QoL) in patients with early psychosis remain relatively unexplored. We evaluated the predictive value of psychopathological and metabolic parameters on QoL in antipsychotic-naïve patients with first-episode psychosis before and after initial antipsychotic treatment. At baseline, 125 patients underwent assessments of psychopathology, prevalence of metabolic syndrome (MetS), and QoL. After 6 weeks of antipsychotic monotherapy, 89 patients were re-investigated. At baseline, the prevalence of MetS was 19.3% (*n* = 22). After 6 weeks, body weight (1.3 kg, *p* < 0.001) and body mass index (0.4 kg/m^2^, *p* < 0.001) increased, and four additional patients developed MetS. Multivariate linear regression revealed that positive and negative symptoms, and to some degree waist circumference, were predictors of QoL at both time points. Our findings suggest that in the earliest stages of antipsychotic treatment, metabolic side-effects may be less influential on QoL than psychopathological severity.

## Introduction

Quality of life (QoL) is defined by the World Health Organization (WHO) as “individuals’ perceptions of their position in life in the context of the culture and value systems in which they live, and in relation to their goals, expectations, standards, and concerns“^1^. Traditionally, studies and treatment of patients with schizophrenia have focused on reducing the burdensome and distressing psychotic symptoms such as hallucinations, formal thought disorders, and delusions^2^. However, following the deinstitutionalization movement during the 1960s and 1970s, it became evident that care for people with severe mental illness living in the community should also consider real-life functioning and QoL^3^. Moreover, it has become increasingly recognized that the less apparent negative symptoms such as apathy, amotivation, and social isolation have a strong predictive impact on the course of illness^4^.

Antipsychotic medication has well-documented ameliorating effects on positive symptoms, however, the effect on negative symptoms is less consistent. Additionally, antipsychotics are potent drugs and side-effects such as weight gain, sexual problems, and dysmetabolism are common clinical challenges^5,6^. Adding to this complexity is the fact that genetic, environmental, and lifestyle factors, also predispose patients with schizophrenia to have and/or develop metabolic problems such as reduced insulin sensitivity and increased body weight even before exposure to antipsychotics^7,8^.

Metabolic syndrome (MetS) is a cluster of symptoms, including central obesity, dyslipidemia, elevated blood pressure, and hyperglycemia, which is associated with an increased risk of cardiovascular disease^9^. The prevalence of MetS in patients with first-episode psychosis (FEP) is estimated at approximately 10%^10^. In comparison, the prevalence of MetS in healthy, young adults is estimated at 7%^11^. Furthermore, young and antipsychotic-naïve patients with FEP are identified to be particularly susceptible to weight gain after initiation of antipsychotic treatment^12^.

The influence of metabolic comorbidities on QoL has mostly been studied in patients with chronic schizophrenia, where weight gain and increased body mass index (BMI) have been related to poorer QoL in this vulnerable population^13,14^. For patients with FEP, a recent pilot study found that QoL appeared unaffected by antipsychotic-related side-effects^15^, however, the small sample size impedes firm conclusions to be drawn. Furthermore, studies of patients with FEP have shown that negative symptoms may be more related to poor QoL than positive symptoms, and that a longer duration of untreated psychosis is associated with reduced QoL^16^.

The aim of this study was two-fold: first, we identified potential psychopathological and metabolic predictors of QoL in a sample of initially antipsychotic-naïve patients with FEP. Second, we investigated the temporal stability of this baseline pattern by including 6-week follow-up data, after patients had undergone initial antipsychotic monotherapy. We hypothesized, that before treatment, symptom severity would be the strongest predictor of QoL, but at follow-up, the antipsychotic-induced metabolic side-effects would be equally strong predictors of QoL.

## Material and methods

The data were derived from two consecutive cohorts of antipsychotic-naïve patients with FEP; the Pan European Collaboration Antipsychotic-naïve Studies (PECANS) cohorts I (*n* = 67) from 2008–2014 and II (*n* = 69) from 2014-2019. Detailed descriptions of the studies can be found in refs. ^17,18^ and www.clinicaltrials.gov (NCT01154829, NCT02339844). Patients provided oral and written informed consent prior to inclusion, and both studies were approved by the Regional Committee on Biomedical Research Ethics (H-D-2008-088, H-3-2013-149).

### Patients

Patients between 18–45 years of age were recruited from hospitals and psychiatric out-patient mental health clinics in the Capital Region of Denmark. Diagnoses according to the International Classification of Diseases 10th revision (ICD-10) were verified using the Schedules for Clinical Assessment in Neuropsychiatry (SCAN), version 2.1^19^. In the PECANS I cohort, patients met the criteria for schizophrenia (DF20.x) or schizoaffective psychosis (DF25.x). In the PECANS II cohort, patients with diagnoses in the non-affective psychotic spectrum (DF2x), except schizotypal disorder (DF21), were also included. Previous exposure to antipsychotic medication or methylphenidate were exclusion criteria. Treatment with antidepressant medication or mood stabilizers more than one month before baseline examinations was accepted. Previous or present use of benzodiazepines was allowed. Other exclusion criteria were current diagnosis of drug dependency, severe physical illness, and involuntary hospitalization or treatment. Current recreational substance use was accepted. In the current project, we included patients, who had provided data on QoL and had undergone assessments covering psychopathology and somatic screening for the presence of MetS. Data were included from baseline and from a 6-week follow-up visit, where patients had undergone initial antipsychotic monotherapy. MetS was defined according to the International Diabetes Federation criteria (Box 1)^20^.

Additionally, data from healthy controls without mental illness (HCs) were used in descriptive post hoc analyses, where we compared baseline BMI between patients with FEP and HCs (Supplementary material, Fig. S1). The data was derived from the Function and Overall Cognition in Ultra-high-risk States (FOCUS) trial with a detailed description being available at www.clinicaltrials.gov (NCT02098408). HCs were 18–40 years and recruited through the internet (www.forsøgsperson.dk) and community-based advertising. A detailed description of the HCs can be found in ref. ^21^.

Box 1 The International Diabetes Federation metabolic syndrome world-wide definition

**Table:** 

Central obesity (defined as waist circumference ≥94 cm for Europid males and ≥80 cm for Europid females or a body mass index (BMI) > 30 kg/m^2^) plus any two of the following:	
• Raised triglycerides	Triglycerides ≥1.7 mmol/L or specific treatment for this lipid abnormality
• Reduced HDL-cholesterol	HDL-cholesterol <1.03 mmol/L in males and <1.29 mmol/L in females or specific treatment for this lipid abnormality
• Raised blood pressure	Systolic blood pressure ≥130 mmHg or Diastolic blood pressure ≥ 85 mmHg or treatment of previously diagnosed hypertension
• Raised fasting plasma glucose	Fasting plasma glucose ≥5.6 mmol/L or previously diagnosed Type 2 diabetes

### Instruments and measures

#### Quality of life

QoL was assessed using a 21-item self-report satisfaction with life scale (SWLS), an ordinal scale measuring current subjective life satisfaction on four domains: living situation, social relationships, self and present life, and work. Each item is scored on a Likert-type 5-point scale ranging from 0 (not at all) to 4 (a great deal) with higher scores reflecting better QoL. Since a confirmatory factor analysis of the scale has been conducted, showing that three items were less than optimal indicators of the underlying domain^22^, we excluded these three items to adapt our version to the validated 18-item SWLS. The 18-item SWLS has been validated for measuring QoL in patients with schizophrenia^22,23^.

#### Psychopathology

Psychopathology was assessed using the positive and negative syndrome scale (PANSS), a 30-item semi-structured interview that evaluates the severity of positive (PANSS-P) and negative (PANSS-N) symptoms as well as general psychopathology (PANSS-G)^24^. PANSS-P and PANSS-N include 7 items each, and PANSS-G includes 16 items. Each subscale is rated on an ordinal scale ranging from 1 to 7 with 7 representing extreme severity. PANSS interviews were carried out by trained medical doctors or nurses and interviews were video-recorded for validation purposes. For the analyses in the current study, we focused on the PANSS-P and PANSS-N subscales.

#### Metabolic syndrome

All patients underwent broad somatic screening to assess the prevalence of MetS. Blood samples were acquired in the morning under fasting conditions. Blood samples were analyzed at the Department of Clinical Biochemistry at the Copenhagen University Hospital Glostrup and were destroyed after analyses. Blood pressure was assessed as a single, seated, and resting measurement. To assess central obesity, waist circumference was measured in a horizontal plane, midway between the inferior margin of the ribs and the superior border of the iliac crest. Height (cm) and body weight (kg) were measured to calculate BMI (kg/m^2^). BMI categories were defined according to WHO cutoff criteria as underweight (BMI < 18.5 kg/m^2^), normal weight (18.5–24.9 kg/m^2^), pre-obesity (25.0–29.9 kg/m^2^), and obesity class I (30.0–34.9 kg/m^2^), class II (35.0–39.9 kg/m^2^) and class III (≥40.0 kg/m^2^)^25^.

### Medication

After completion of baseline assessments, patients were treated for 6 weeks with amisulpride in PECANS I and aripiprazole in PECANS II. Amisulpride is primarily a D2/3 receptor antagonist, whereas aripiprazole among others acts as a partial D2 receptor agonist. In both cohorts, patients were treated with individual doses and monitored closely to balance clinical effects and side effects. In order to compare doses of both cohorts, chlorpromazine equivalent doses were calculated using a conversion factor of 0.86 for amisulpride, and a conversion factor of 20.0 for aripiprazole^26^.

### Statistics

Statistical significance was defined as *p*-value < 0.05, two-sided. To account for multiple comparisons, *p*-values were corrected using the false-discovery rate (FDR)^27^.

#### Descriptive statistics

Continuous variables were reported as mean and standard deviation (SD) or as median and range depending on the distribution of data. Categorical variables were reported as frequency and percentage. To examine differences in clinical measures between baseline and 6-week follow-up, paired samples t-tests or Wilcoxon tests were applied to continuous variables as applicable, and the Chi-square tests were applied for categorical variables. To investigate whether the drop in baseline size impacted the results, we conducted identical statistical tests, but with analyses restricted to patients with data at both time points (i.e., *N* = 89). To evaluate the differences between patients who completed the study and patients who dropped out, we compared baseline variables on demography, psychopathology, and metabolic measures between the two groups using independent samples *t*-tests or Mann–Whitney *U*-test depending on the distribution of data for continuous variables, and Chi-square tests for categorical variables.

#### Multivariate linear regression analyses

To identify the pattern between our independent and dependent variables (i.e., potential predictors of QoL), multivariate linear regression analyses were conducted as our main analyses. With psychopathological and metabolic measurements as our independent variables, a series of models were constructed, each with a different SWLS subdomain as the dependent variable. A forward selection method, using the R package “olsrr” (version 0.5.3), specifically the "ols_step_forward_aic" function^28^, was applied. In a stepwise manner, potential independent variables that decreased akaike information criterion (AIC) of the model were included one by one, and the final model was selected when the remaining independent variables no longer decreased the AIC value of the model. All models were adjusted for age and sex. Only patients with a complete dataset were included in the regression analysis. Residuals were further tested for homoscedasticity, independence, and normality. The variance inflation factor never exceeded three in any of the final models, indicating that multi-collinearity was not a concern.

## Results

### Patients

**Fig. 1:**
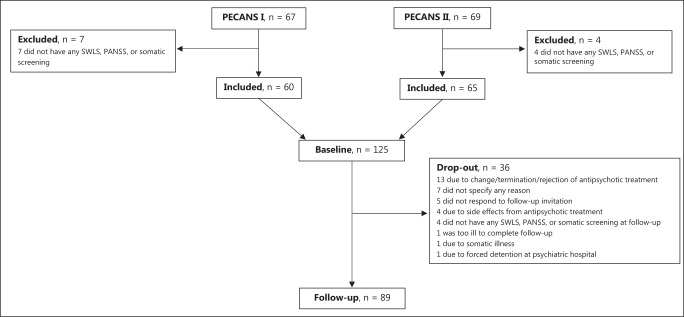
Flowchart. SWLS Satisfaction with life scale, PANSS Positive and negative syndrome scale.

Demographic and clinical characteristics are presented in Table 1. At baseline, 125 antipsychotic-naïve patients (58 females) with FEP were included, and after 6 weeks, 89 patients (42 females) were re-investigated. Numbers and reasons for exclusion and drop-outs are illustrated in Fig. 1. After 6 weeks, patients in PECANS I received a median daily dosage of 200 mg amisulpride (25–75th percentile = 150–300 mg; range = 0–800 mg), and patients in PECANS II received a median daily dosage of 10.0 mg aripiprazole (25–75th percentile = 5.0–15.0 mg; range = 2.5–25.0 mg) (Table 2). No differences were observed when comparing the baseline and follow-up group only including patients with data at both time-points, except for sex, MetS, and triglycerides (Supplementary material, Table S1). Additionally, no differences were found in baseline values of patients who completed the study and patients who dropped out (Supplementary material, Table S2). Several patients did not respond to the items comprising the work subdomain (i.e., items 12 and 13) of SWLS at both baseline (*n* = 27) and follow-up (*n* = 15) (Table 1). At baseline, most of these patients were either unemployed or students, and these circumstances did not change during the 6 weeks. As a result, we excluded the work subdomain from further analyses (i.e., two more items were excluded in addition to the three already excluded).

**Table 1:** Characteristics of included antipsychotic-naïve patients with first-episode psychosis.

Variables	Values				
		Baseline		6-week follow-up	*p-*value
	*n*	*(N* = 125)	*n*	(*N* = 89)	
Age, years	125	22.7 [18.2; 42.6]			
Female^a^ (*n* (%))	125	58 (46)	89	42 (47)	0.945
*Satisfaction with life scale*					
Living situation^c^	125	7.9 (3.4)	89	8.6 (3.6)	0.288
Social relationships^b^	124	10.2 (5.4)	89	12.5 (5.0)	**<0.001**
Self and present life^b^	120	6.9 (4.3)	89	10.6 (4.8)	**<0.001**
Work^c^	98	2.5 [0.0; 8.0]	74	3 [0; 8]	0.234
PANSS total^b^	125	78.2 (15.5)	89	62.2 (13.8)	**<0.001**
PANSS-P^b^	125	19.2 (4.1)	89	14.1 (3.8)	**<0.001**
PANSS-N^b^	125	19.8 (6.3)	89	17.6 (5.7)	**0.003**
PANSS-G^b^	125	39.2 (8.3)	89	30.6 (7.5)	**<0.001**
MetS^a,d^ (*n* (%))	114	22 (19.3)	80	14 (17.5)	0.839
Sum of met IDF criteria^c,d^	22	3.0 [3.0; 5.0]	14	3.5 (3.0; 5.0)	0.613
Body weight, kg^b^	125	70.6 [39.0; 143.0]	88	71.1 [40.4; 134.5]	**<0.001**
Body mass index, kg/m^2 b^	124	23.4 [16.3; 49.5]	88	23.4 [16.5; 45.5]	**<0.001**
Waist circumference, cm^b^	122	80 [58; 127]	84	82 [64; 124]	0.060
Systolic blood pressure, mmHg^b^	125	127 [101; 185]	88	123 [101; 164]	**0.002**
Diastolic blood pressure, mmHg^b^	125	78 (9)	88	77 (9)	0.061
Fasting plasma glucose, mmol/L^c^	115	5.1 [3.8; 6.8]	79	5.2 [4.0; 6.9]	0.288
Triglycerides, mmol/L^c^	113	1.0 [0.3; 4.9]	81	1.0 [0.4; 4.4]	0.945
High-density lipoprotein cholesterol, mmol/L^b^	114	1.2 [0.6; 2.8]	81	1.3 [0.7; 2.5]	0.633

Descriptive statistics was reported as mean (SD) or median [range] depending on the distribution of data. Categorical variables were reported as frequency (%).

*PANSS* Positive and Negative Syndrome Scale, *PANSS-P* positive symptoms, *PANSS-N* negative symptoms, *PANSS-G* general psychopathology, MetS metabolic syndrome, *IDF* International Diabetes Federation.

^a^Chi-square test.

^b^Paired samples *t*-test.

^c^Wilcoxon test.

^d^According to the International Diabetes Federation.

*p*-values shown in bold survived false-discovery rate (FDR) correction.

**Table 2:** Psychopharmacological treatment at 6-week follow-up.

		Amisulpride		Aripiprazole
		(PECANS I, *N* = 41)		(PECANS II, *N* = 48)
Dose (mg)	*n* = *41*	200 [0–800]	*n* = *47*	10.0 [2.5–25.0]
CPZ-equivalent dose (mg)		171 [0–686]		200 [50–500]

Doses reported as median [range].

After 6 weeks, improvements were seen for QoL in the subdomains social relationships of 2.3 units (95%CI: 1.5 to 3.1, *p* < 0.001) and self and present life of 3.7 units (95%CI: 2.8 to 4.6, *p* < 0.001), and for psychopathology in both PANSS total of –14.7 units (95%CI: –17.5 to –11.9, *p* < 0.001), PANSS-P of –5.0 units (95%CI: –5.9 to –4.1, *p* < 0.001), PANSS-N of 1.9 units (95%CI: –3.1 to –0.8, *p* = 0.002), and PANSS-G of –7.8 units (95%CI: –9.3 to –6.2, *p* < 0.001). Systolic blood pressure decreased during the 6 weeks with –4 mm Hg (95%CI: –6 to –2, *p* = 0.002). Increases were observed in body weight of 1.3 kg (95%CI: 0.7 to 1.9; *p* < 0.001) and BMI of 0.4 kg/m^2^ (95%CI: 0.2 to 0.6; *p* < 0.001). Patients with sufficient data to comply with IDF criteria for MetS (*n* = 114 at baseline and *n* = 80 at follow-up) were evaluated for the presence of MetS. At baseline, the prevalence of MetS was 19.3% (*N* = 22). After 6 weeks, 15 of these patients were re-assessed and ten of these patients still met the criteria for MetS. Additionally, four patients had developed MetS during the 6 weeks. Finally, descriptive post hoc analyses at baseline showed that the number of patients in the pre-obesity/obesity BMI category was ~2-fold higher compared to healthy controls (Supplementary material, Fig. S1).

### Multivariate linear regression analyses: predictors of quality of life

We conducted multivariate linear regression analyses to identify potential predictors of living situation, social relationships, and self and present life at baseline and follow-up. At baseline, Model 1 explained 12% of the variance in living situation (adjusted *R*^2^ = 0.123), with PANSS-P and waist circumference being significant predictors (*β* = –0.222, *p* = 0.002 and *β* = –0.065, *p* = 0.031, respectively). Model 2 and Model 3 explained 20% of the variance in social relationships (adjusted *R*^2^ = 0.198), and 10% of the variance in self and present life (adjusted *R*^2^ = 0.098), respectively, with PANSS-N being a significant predictor (*β* = –0.312, *p* < 0.001 and *β* = –0.167, *p* = 0.015, respectively) (summarized in Table 3). These patterns remained after 6 weeks (Supplementary material, Table S3). Additionally, at baseline, age was found to be a significant predictor of social relationships (*β* = –0.217, *p* = 0.010), and diastolic blood pressure of self and present life (*β* = 0.137, *p* = 0.008). However, these associations were not observed after 6 weeks.

**Table 3:** Multivariate linear regression | Baseline.

Dependent variables	Model 1 Living situation			Model 2 Social relationships			Model 3 Self and present life		
Independent variables^a^	*β*	SE	*p*	*β*	SE	*p*	*β*	SE	*p*
PANSS-P	–0.222	0.069	**0.002**						
PANSS-N				−0.312	0.073	**<0.001**	–0.167	0.067	**0.015**
Sum of met IDF criteria	0.605	0.331	0.071						
Waist circumference, cm	–0.065	0.030	**0.031**						
Diastolic blood pressure, mmHg							0.137	0.051	**0.008**
Sex^b^	0.744	0.616	0.230	–1.246	0.927	0.182	0.429	0.848	0.614
Age, years	–0.050	0.056	0.375	–0.217	0.082	**0.010**	–0.013	0.078	0.874
Constant	7.788	0.429	**<0.001**	10.893	0.658	**<0.001**	6.898	0.607	**<0.001**
Number of observations	108			107			104		
*R*^2^	0.164 (16%)			0.218 (22%)			0.133 (13%)		
Adjusted *R*^2^	0.123 (12%)			0.195 (20%)			0.098 (10%)		
*F*	4.006			9.578			3.800		
*p*	**0.002**			**<0.001**			**0.006**		

*PANSS* Positive and Negative Syndrome Scale, *PANSS-P* positive symptoms, *PANSS-N* negative symptoms, *IDF* International Diabetes Federation.

^a^Continuous variables are mean-centered.

^b^Dummy-coded variables: female = −1, male = 1.

Significant *p*-values shown in bold.

## Discussion

In this study, we aimed to identify potential psychopathological and metabolic predictors of quality of life in a sample of initially antipsychotic-naïve patients with first-episode psychosis. Our main findings are that positive and negative symptoms are significant predictors of quality of life before initiation of antipsychotic treatment. Specifically, positive symptoms were a predictor of satisfaction with living situations, whereas negative symptoms were a predictor of satisfaction with social relationships and self and present life. This supports the first term of our hypothesis, that symptom severity would be the strongest predictor of quality of life before treatment. Second, we investigated the temporal stability of this baseline pattern by including 6-week follow-up data, after patients had undergone initial antipsychotic monotherapy. Even though 6 weeks of antipsychotic treatment reduced psychopathology, symptom severity remained the strongest predictor of quality of life at follow-up. Apart from the vague predictive value of waist circumference on satisfaction with living situation, we found no associations between quality of life and metabolic measurements at follow-up, thus disproving the second term of our hypothesis that antipsychotic-induced metabolic side-effects would be an equally strong predictor of quality of life after 6 weeks of antipsychotic monotherapy.

To the best of our knowledge, this is the first study to investigate the predictive value of psychopathological and metabolic parameters on quality of life in a cohort of antipsychotic-naïve patients with first-episode psychosis before and after initial antipsychotic treatment. Our data confirm that psychopathology is a key factor relating to quality of life in first-episode psychosis. Specifically, the results extend previous findings identifying the strong association between negative symptoms and quality of life^16,29^. This association supports an overlap between the subdomain social relationships and the negative symptoms subscale that contains items measuring emotional withdrawal and passive/apathetic social withdrawal. Further, we confirm that also positive symptoms impair quality of life which is in line with several^30–32^, but not all previous studies^33^.

The baseline prevalence of metabolic syndrome in our sample was 19.3% (*n* = 22) which is higher compared to previous reports^10,34,35^. Our post hoc analyses revealed that the proportion of patients in our population with pre-obesity or obesity was ~2-fold higher compared to healthy controls (Supplementary material, Fig. S1). This may partly reflect the consistent increases in the prevalence of pre-obesity and obesity in European countries^36^, however, with a higher variety of weight distribution in our patient population, i.e., higher proportions represented in the extremes (i.e., underweight, obesity class I, II, and III). In the present study, we were unable to make a direct comparison of the prevalence of metabolic syndrome between patients and healthy controls. However, a long-term study found distinct differences in the metabolic profile at baseline along with considerable exacerbation of metabolic disturbances in patients compared to healthy controls during the follow-up period, including higher rates of obesity and metabolic syndrome^37^. Overall, these observations highlight the intimate link between psychotic disorders and the increased risk of somatic comorbidities.

In the current study, quality of life appeared unaffected by the presence of metabolic syndrome both prior to and after initiation of antipsychotic treatment, which is in line with the previously mentioned pilot study^15^. These results indicate that in the earliest stages of antipsychotic treatment, metabolic side-effects may be less influential on quality of life than psychopathological severity. It is important to note that neither amisulpride nor aripiprazole are among the antipsychotics which are most strongly associated with metabolic side effects. However, both drugs are frequently prescribed in clinical practice, especially in this patient population being antipsychotic-naïve. Furthermore, young, and antipsychotic-naïve patients are generally more sensitive to the development of antipsychotic-induced weight gain, and this may occur with both amisulpride and aripiprazole^38,39^.

Systolic blood pressure decreased from baseline to follow-up. However, it should be noted that blood pressure was only performed as a single measure at both time points increasing the risk of measurement bias. Moreover, enhanced familiarity between patients and the assessment environment, as well as the reduction in the severity of psychopathological symptoms, may have decreased the adrenergic response which in turn may have contributed to reduced blood pressure at follow-up.

We believe that our findings have important clinical implications. Psychopathological severity appears to be crucial in this early illness phase, however, emerging associations between central obesity and quality of life are also evident. Overall, this justifies the importance of early patient involvement in treatment decision-making, aligning it more closely with patients’ preferences and desires.

### Strengths and limitations

The major strength of the present study is our unique sample of antipsychotic-naïve patients with first-episode psychosis whom we followed after initial antipsychotic exposure. However, our modest sample size may be considered a limitation, and therefore, generalization of the results should be done with caution. Second, our intervention period was limited to 6 weeks, which may be insufficient to invoke changes that would affect quality of life. Nonetheless, the chosen time period was based on several considerations, notably the main goal of investigating the early predictors of quality of life, specifically in this initial and critical phase of illness. The short follow-up period enabled us to investigate emerging changes in quality of life, and specifically, the inclusion of antipsychotic-naïve patients allowed us to examine if changes in quality of life coincided with the introduction of antipsychotic treatment and potential antipsychotic-mediated metabolic side-effects. Second, long-term studies involve the risk of medication shifts or even non-compliance. In our study, this risk is minimized by following the patients closely over a short period of time, and ultimately, enhances the integrity and strength of data. Third, the absence of complete comparable data in healthy controls impedes inferences as to whether metabolic markers are also associated with quality of life in the background population.

## Conclusion

In summary, we confirmed that psychopathological severity is directly impacting the quality of life both before and after treatment. Moreover, in the earliest stages of antipsychotic treatment, metabolic side effects may be less influential on quality of life than psychopathological severity. Finally, our findings underline the intimate link between psychotic disorders and metabolic aberrations by showing high rates of metabolic syndrome in the antipsychotic-naïve state as well as the aggravation of metabolic parameters already from initial antipsychotic exposure.

### Supplementary information

Table S1

Table S2

Table S3

Figure S1

## Data Availability

Aggregated data from this study are available upon reasonable request to the corresponding author.

## Code availability

All data were analyzed using RStudio statistical software version 4.2.0. The codes used for this study can be accessed upon reasonable request by the corresponding author.
