# Supplementary material for: Influence of psychopathology and metabolic parameters on quality of life in patients with first-episode psychosis before and after initial antipsychotic treatment
Source: Schizophrenia (Heidelb). 2023 Nov 7;9(1):76. doi: 10.1038/s41537-023-00402-8 (PMC10630335; doi:10.1038/s41537-023-00402-8)
Supplement: Supplementary file 1 — Table S1 [file 41537_2023_402_MOESM1_ESM.docx]

| *Table S1.* Characteristics of included antipsychotic-naïve patients with first-episode psychosis, with data at both time-points | | | | | |
| --- | --- | --- | --- | --- | --- |
| *Variables* | *Values* | | | | |
|  |  | Baseline |  | 6-week follow-up | *p-value* |
|  | n | (N = 89) | n | (N = 89) |  |
| Age, years | 89 | 22.6 [18.2; 42.6] |  |  |  |
| Female^a^ (n (%)) | 89 | 42 (47) | 89 | 42 (47) | 1 |
| *Satisfaction with life scale* |  |  |  |  |  |
| Living situation^c^ | 89 | 8.0 (3.3) | 89 | 8.6 (3.6) | 0.288 |
| Social relationships^b^ | 89 | 10.2 (5.4) | 89 | 12.5 (5.0) | **< 0.001** |
| Self and present life^b^ | 86 | 6.9 (4.1) | 89 | 10.6 (4.8) | **< 0.001** |
| Work^c^ | 71 | 3.0 [0.0; 8.0] | 74 | 3.0 [0.0; 8.0] | 0.234 |
| PANSS total^b^ | 89 | 76.9 (15.0) | 89 | 62.2 (13.8) | **< 0.001** |
| PANSS-P^b^ | 89 | 19.1 (4.3) | 89 | 14.1 (3.8) | **< 0.001** |
| PANSS-N^b^ | 89 | 19.5 (6.2) | 89 | 17.6 (5.7) | **0.003** |
| PANSS-G^b^ | 89 | 38.3 (7.6) | 89 | 30.6 (7.5) | **< 0.001** |
| MetS^a,d^ (n (%)) | 81 | 15 (18.5) | 80 | 14 (17.5) | 0.968 |
| Sum of met IDF criteria^c,d^ | 15 | 3.0 [3.0; 5.0] | 14 | 3.5 (3.0; 5.0) | 0.613 |
| Body weight, kg^b^ | 89 | 70.0 [39.0; 132.0] | 88 | 71.1 [40.4; 134.5] | **< 0.001** |
| Body mass index, kg/m^2 b^ | 89 | 23.1 [16.3; 44.6] | 88 | 23.4 [16.5; 45.5] | **< 0.001** |
| Waist circumference, cm^b^ | 87 | 80 [58; 123] | 84 | 82 [64; 124] | 0.060 |
| Systolic blood pressure, mm Hg^b^ | 89 | 126 [104; 185] | 88 | 123 [101; 164] | **0.002** |
| Diastolic blood pressure, mm Hg^b^ | 89 | 78 (9) | 88 | 77 (9) | 0.061 |
| Fasting plasma glucose, mmol/L^c^ | 82 | 5.1 (3.8; 6.3) | 79 | 5.2 [4.0; 6.9] | 0.288 |
| Triglycerides, mmol/L^c^ | 80 | 1.0 [0.3; 4.9] | 81 | 1.0 [0.4; 4.4] | 0.998 |
| High-density lipoprotein cholesterol, mmol/L^b^ | 81 | 1.2 [0.6; 2.8] | 81 | 1.3 [0.7; 2.5] | 0.633 |
| Descriptive statistics was reported as mean (SD) or median [range] depending on the distribution of data. Categorical variables were reported as frequency (%).  ^a^ Chi-square test; ^b^ Paired samples t-test; ^c^ Wilcoxon test  ^d^ According to the International Diabetes Federation.  p values shown in bold survived false-discovery rate (FDR) correction.  PANSS: Positive and negative syndrome scale; PANSS-P: Positive symptoms; PANSS-N: Negative symptoms; PANSS-G: General psychopathology; MetS: Metabolic syndrome; IDF: International Diabetes Federation. | | | | | |
