# Supplementary material for: Influence of psychopathology and metabolic parameters on quality of life in patients with first-episode psychosis before and after initial antipsychotic treatment
Source: Schizophrenia (Heidelb). 2023 Nov 7;9(1):76. doi: 10.1038/s41537-023-00402-8 (PMC10630335; doi:10.1038/s41537-023-00402-8)
Supplement: Supplementary file 2 — Table S2 [file 41537_2023_402_MOESM2_ESM.docx]

| *Table S2.* Baseline characteristics of included antipsychotic-naive patients with first-episode psychosis that completed follow-up visit vs. dropped out | | | | | |
| --- | --- | --- | --- | --- | --- |
|  | | | | | |
| *Variables* | *Values* | | | | |
|  |  | Completed follow-up |  | Dropped out | *p-value* |
|  | n | (N = 89) | n | (N = 36) |  |
| Age, years^c^ | 89 | 22.6 [18.2; 42.6] | 36 | 22.8 [18.3; 37.0] | 0.978 |
| Female^a^ (n (%))^a^ | 89 | 42 (47) | 36 | 16 (44) | 0.978 |
| *Satisfaction with life scale* |  |  |  |  |  |
| Living situation^b^ | 89 | 8.0 (3.3) | 36 | 7.5 (3.6) | 0.935 |
| Social relationships^b^ | 89 | 10.2 (5.4) | 35 | 10.4 (5.5) | 0.978 |
| Self and present life^c^ | 86 | 6 [0; 18] | 34 | 5 [0; 20] | 0.978 |
| Work^c^ | 71 | 3 [0; 8] | 27 | 2 [0; 8] | 0.935 |
| PANSS total^b^ | 89 | 76.9 (15.0) | 36 | 81.3 (16.4) | 0.935 |
| PANSS-P^b^ | 89 | 19.1 (4.3) | 36 | 19.6 (3.8) | 0.935 |
| PANSS-N^b^ | 89 | 19.5 (6.2) | 36 | 20.4 (6.4) | 0.935 |
| PANSS-G^b^ | 89 | 38.3 (7.6) | 36 | 41.3 (9.5) | 0.700 |
| MetS^a,d^ (n (%)) | 81 | 15 (16.9) | 33 | 7 (19.4) | 0.978 |
| Sum of met IDF criteria^c,d^ | 15 | 3 [3; 5] | 7 | 3 [3; 3] | 0.700 |
| Body weight, kg^c^ | 89 | 70 [39; 132] | 36 | 70.8 [40.0; 143.0] | 0.978 |
| Body mass index, kg/m^2 c^ | 89 | 23.1 [16.3; 44.6] | 35 | 24.4 [17.7; 49.5] | 0.935 |
| Waist circumference, cm^c^ | 87 | 80 [58; 123] | 35 | 81 [60; 127] | 0.935 |
| Systolic blood pressure, mm Hg^c^ | 89 | 126 [104; 185] | 36 | 130 [101; 164] | 0.935 |
| Diastolic blood pressure, mm Hg^b^ | 89 | 78 (9) | 36 | 77 (9) | 0.978 |
| Fasting plasma glucose, mmol/L^c^ | 82 | 5.1 [3.8; 6.3] | 33 | 5.1 [4.4; 6.8] | 0.978 |
| Triglycerides, mmol/L^c^ | 80 | 1.0 [0.3; 4.9] | 33 | 0.9 [0.6; 3.8] | 0.978 |
| High-density lipoprotein cholesterol, mmol/L^c^ | 81 | 1.2 [0.6; 2.8] | 33 | 1.2 [0.7; 1.8] | 0.935 |
| Descriptive statistics was reported as mean (SD) or median [range] depending on the distribution of data. Categorical variables were reported as frequency (%).  ^a^ Chi-square test; ^b^ independent samples t-test; ^c^ Mann-Whitney-U test ^d^ According to the International Diabetes Federation.  All p values were adjusted using the false-discovery rate (FDR).  PANSS: Positive and negative syndrome scale; PANSS-P: Positive symptoms; PANSS-N: Negative symptoms; PANSS-G: General psychopathology; MetS: Metabolic syndrome; IDF: International Diabetes Federation. | | | | | |
