# Supplementary material for: Influence of psychopathology and metabolic parameters on quality of life in patients with first-episode psychosis before and after initial antipsychotic treatment
Source: Schizophrenia (Heidelb). 2023 Nov 7;9(1):76. doi: 10.1038/s41537-023-00402-8 (PMC10630335; doi:10.1038/s41537-023-00402-8)
Supplement: Supplementary file 3 — Table S3 [file 41537_2023_402_MOESM3_ESM.docx]

| *Table S3.* Multivariate linear regression \| Follow-up | | | | | | | | | | | | | | | | | |
| --- | --- | --- | --- | --- | --- | --- | --- | --- | --- | --- | --- | --- | --- | --- | --- | --- | --- |
|  | *Dependent variables* | |  | Model 4  **Living situation** | | |  | | Model 5 **Social relationships** | | |  | | Model 6  **Self and present life** | | | |
| *Independent variables*^a^ | |  | | β | SE | *p* |  | β | | SE | *p* | |  | | β | SE | *p* |
| PANSS-P | |  | | -0.325 | 0.100 | **0.002** |  |  | |  |  | |  | |  |  |  |
| PANSS-N | |  | |  |  |  |  | -0.348 | | 0.092 | **< 0.001** | |  | | -0.265 | 0.093 | **0.005** |
| Sum of met IDF criteria | |  | | 0.702 | 0.384 | 0.072 |  |  | |  |  | |  | |  |  |  |
| Waist circumference, cm | |  | | -0.074 | 0.036 | **0.043** |  |  | |  |  | |  | |  |  |  |
| Fasting plasma glucose, mmol/L | |  | |  |  |  |  | -2.048 | | 1.163 | 0.082 | |  | |  |  |  |
| Triglycerides, mmol/L | |  | |  |  |  |  | 1.370 | | 0.920 | 0.141 | |  | |  |  |  |
| Sex^b^ | |  | | 0.206 | 0.825 | 0.804 |  | -0.666 | | 1.195 | 0.579 | |  | | -1.234 | 1.110 | 0.270 |
| Age, years | |  | | -0.053 | 0.072 | 0.457 |  | -0.195 | | 0.103 | 0.063 | |  | | -0.042 | 0.097 | 0.666 |
| Constant | |  | | 8.626 | 0.581 | **< 0.001** |  | 12.899 | | 0.834 | **< 0.001** | |  | | 11.560 | 0.803 | **< 0.001** |
| Number of observations | |  | | 76 |  |  |  | 76 | |  |  | |  | | 76 |  |  |
| R^2^ | |  | | 0.214 (21%) |  |  |  | 0.272 (27%) | |  |  | |  | | 0.130 (13%) |  |  |
| Adjusted R^2^ | |  | | 0.158 (16%) |  |  |  | 0.220 (22%) | |  |  | |  | | 0.094 (9%) |  |  |
| *F* | |  | | 3.815 |  |  |  | 5.239 | |  |  | |  | | 3.593 |  |  |
| *p* | |  | | **0.004** |  |  |  | **< 0.001** | |  |  | |  | | **0.018** |  |  |
| ^a^ Continuous variables are mean-centered. ^b^ Dummy-coded variables: female = -1, male = 1  PANSS: Positive and negative syndrome scale; PANSS-P: Positive symptoms; PANSS-N: Negative symptoms; IDF: International Diabetes Federation.  Significant *p*-values shown in bold. | | | | | | | | | | | | | | | | | |
