# Supplementary material for: Influence of psychopathology and metabolic parameters on quality of life in patients with first-episode psychosis before and after initial antipsychotic treatment
Source: Schizophrenia (Heidelb). 2023 Nov 7;9(1):76. doi: 10.1038/s41537-023-00402-8 (PMC10630335; doi:10.1038/s41537-023-00402-8)
Supplement: Supplementary file 4 — Figure S1 [file 41537_2023_402_MOESM4_ESM.pdf]

Figure S1. Healthy controls vs. antipsychotic-naïve patients with first episode psychosis according to BMI categories | Baseline

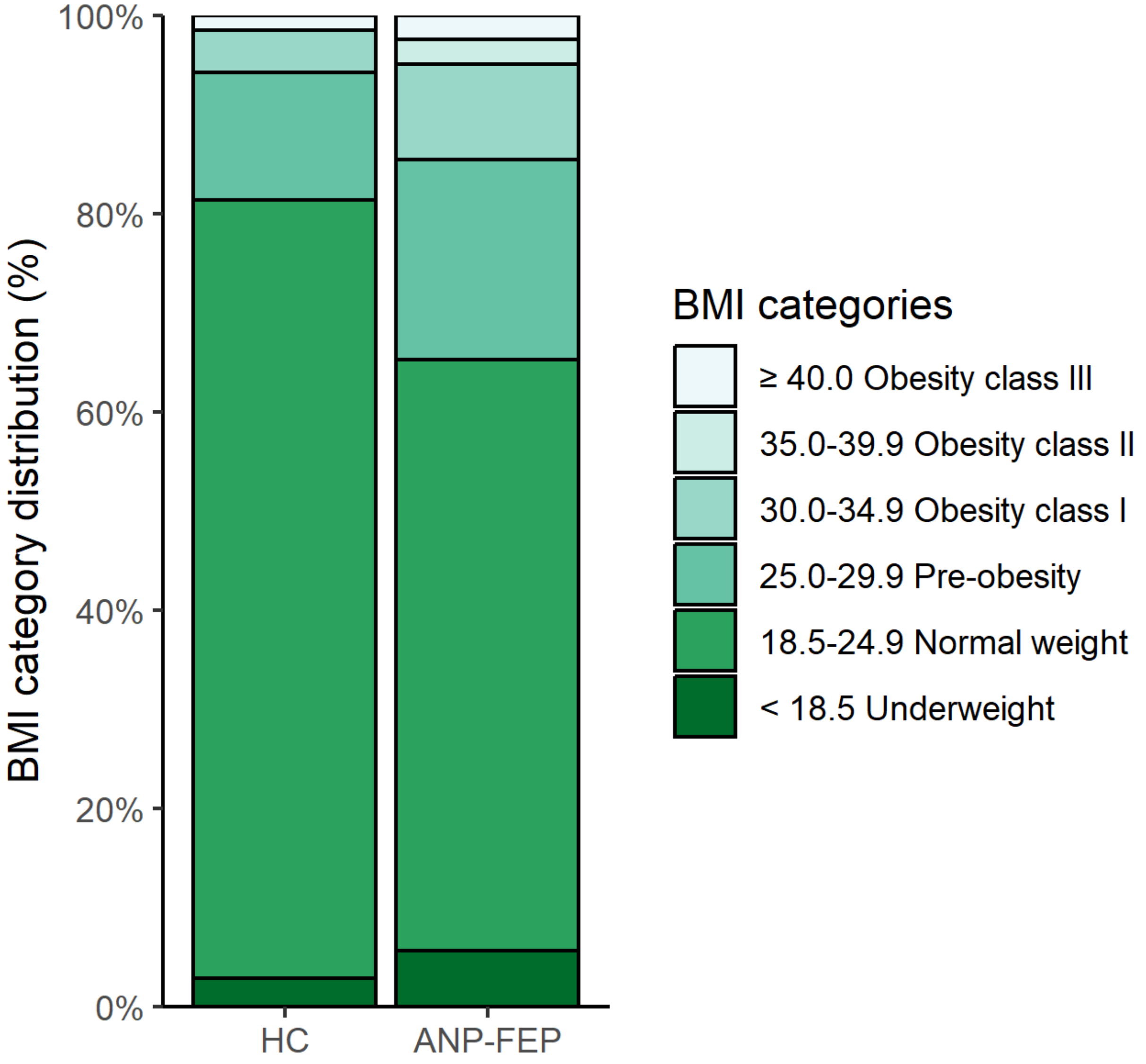

Exclusion criteria for HCs were IQ < 70 or a documented history of developmental delay or intellectual disability, severe medical condition, previous head injury with unconsciousness > 5 minutes, previous or current psychiatric diagnosis, including substance abuse/dependency, and first degree relative with a psychotic disorder.
HC: Healthy controls; BMI: Body mass index; ANP-FEP: Antipsychotic-naïve patients with first-episode psychosis.
